# Unusual patterns of hybridization involving two alpine *Salvia* species: Absence of both F_1_ and backcrossed hybrids

**DOI:** 10.3389/fpls.2022.1010577

**Published:** 2022-10-18

**Authors:** Yuhang Chang, Shengxuan Zhao, Hanwen Xiao, Detuan Liu, Yanbo Huang, Yukun Wei, Yongpeng Ma

**Affiliations:** ^1^ Yunnan Key Laboratory for Integrative Conservation of Plant Species with Extremely Small Populations, Kunming Institute of Botany, Chinese Academy of Sciences, Kunming, China; ^2^ University of Chinese Academy of Sciences, Beijing, China; ^3^ College of Traditional Chinese Medicine, Tianjin University of Traditional Chinese Medicine, Tianjin, China; ^4^ Eastern China Conservation Center for Wild Endangered Plant Resources, Shanghai Chenshan Botanical Garden, Shanghai, China; ^5^ Shanghai Botanical Garden, Shanghai, China

**Keywords:** hybridization, *Salvia*, RAD-seq, speciation, reproductive isolation barriers, ethological isolation

## Abstract

Natural hybridization plays an important role in speciation; however, we still know little about the mechanisms underlying the early stages of hybrid speciation. Hybrid zones are commonly dominated by F_1_s, or backcrosses, which impedes further speciation. In the present study, morphological traits and double digest restriction‐site associated DNA sequencing (ddRAD-seq) data have been used to confirm natural hybridization between *Salvia flava* and *S*. *castanea*, the first case of identification of natural hybridization using combined phenotypic and molecular evidence in the East Asian clade of *Salvia*. We further examined several reproductive barriers in both pre-zygotic and post-zygotic reproductive stages to clarify the causes and consequences of the hybridization pattern. Our results revealed that reproductive isolation between the two species was strong despite the occurrence of hybridization. Interestingly, we found that most of the hybrids were likely to be F_2_s. This is a very unusual pattern of hybridization, and has rarely been reported before. The prevalence of geitonogamy within these self-compatible hybrids due to short distance foraging by pollinators might explain the origin of this unusual pattern. F_2_s can self-breed and develop further, therefore, we might be witnessing the early stages of hybrid speciation. Our study provides a new case for understanding the diversification of plants on the Qinghai-Tibet Plateau.

## 1 Introduction

Where two recently diverged plant species come into secondary contact in a part of their distribution areas, two forces control their behavior: fusion by hybridization-mediated gene flow, and separation by reproductive isolation (RI) formed during the early speciation stage before their secondary contact (Rieseberg and Carney, 1998; Hewitt, 2001; Abbott et al., 2013). Therefore, various patterns of hybridization might occur if RI varied between parental plants and their hybrids. For instance, hybrid zones dominated by F_1_s have been repeatedly detected cross many plant genera and families in recent years (Milne et al., 2003; Zha et al., 2010; Liao et al., 2015; Zhang et al., 2018; Hu et al., 2021a; Hu et al., 2021b; Liao et al., 2021), indicating that gene flow is fully blocked and RI is nearly complete between parent species. Hybrid zones dominated by F_1_s are usually due to F_1_s having higher habitat-mediated superiority than other genotype classes or a high degree of hybrid sterility (Milne et al., 2003; Yu et al., 2011). In this pattern, the elimination of post-F_1_ hybrid derivatives actually prevent gene flow and then strengthen the boundaries of parental species. Another common circumstance is where the hybrid zone is dominated by backcrosses, which occurs when F_1_s are surrounded by parental plants and cross-pollination between them could occur frequently, and may lead to introgression and/or fusion between parental species (Lepais et al., 2009; Arnold et al., 2010; Ma et al., 2014; Balao et al., 2015; Ma et al., 2019).

Due to the unique staminal lever mechanism, *Salvia* L. has become a famous model for the study of interactions between pollinators and plants (Claβen-Bockhoff et al., 2003), and the trait is thought to be a key factor in the isolation of different *Salvia* species (Walker and Sytsma, 2007). For example, *S. liguliloba* and *S. bowleyana* share pollinators (bumblebee) in sympatric populations, however, differences in lever structure allow pollen to be deposited on different parts of the bumblebee’s body and thus avoid hybridization (Wei et al., 2017). Although different flower structures in different *Salvia* species can lead to a large RI (Walker and Sytsma, 2007), there are still many reports of natural hybridization in different clades. These cases are mostly described based on morphology (Epling, 1938; Epling, 1947; Anderson and Anderson, 1954; Grant and Grant, 1964; Webb and Carlquist, 1964; Meyn, 1987; Hihara et al., 2001; Wood, 2014; Rivera et al., 2019), and only a few studies have relied on molecular evidence (Walker et al., 2015; Radosavljević et al., 2019; Celep et al., 2020). In addition, in the East Asian *Salvia* clade (i.e., Subgenus *Glutinaria*; Hu et al., 2018), which is a monophyletic lineage, studies on natural hybridization are extremely scarce (Hihara et al., 2001). In particular, to date there has been no strong evidence to verify natural hybridization in this clade.

As an important biodiversity hotspot, the Qinghai-Tibet Plateau (QTP) has reported a large number of natural hybridization events in highly diverse plant genera (Mittermeier et al., 2011; Wu et al., 2022). Therefore, hybridization is likely to be a significant driving force for species radiation in this region. This region is also the center of diversity of the East Asian clade of *Salvia* (Wei et al., 2015). Both *Salvia flava* Forrest ex Diels and *S. castanea* Diels are belonging to the core subclade of Subgen. *Glutinaria* (Hu et al., 2018), distributed in the QTP. During field investigations, we found some individuals with intermediate morphology and concluded that they are putative hybrids between *S. flava* and *S. castanea*. In this study, double digest restriction site-associated DNA sequencing (ddRAD-seq) was performed to clarify this phenomenon, and we aim to (1) confirm the hybridization by molecular evidence; (2) explore the pattern of hybrid zone; and (3) quantify the degree of isolation between parental species through calculations of RI to explain the origin of the pattern of hybridization.

## 2 Materials and methods

### 2.1 Species, study site and sampling

Both *Salvia flava* and *S. castanea* are perennial herbs, and are both diploid (2n = 16; Hu, 2015). *S. flava* is found in Yunnan and Sichuan at elevations of 2500–4000 m, while *S. castanea* has a wider distribution area, including Yunnan, Sichuan, Guizhou and Xizang in China, as well as Nepal and Bhutan, and grows at altitudes between 2500 and 3400 m.

**Figure 1:**
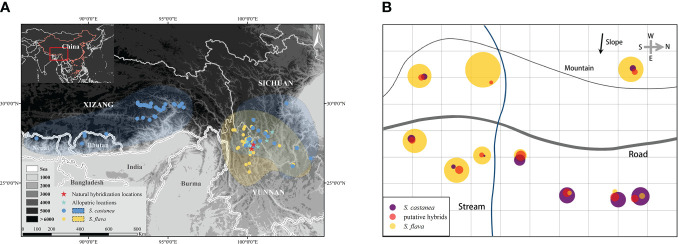
Geographical distribution of *S*. *flava* and *S*. *castanea* **(A)** and the habitat distribution of *S*. *flava*, *S*. *castanea* and hybrids in MY **(B)**. The area of circle represents the number of individuals of the three taxa in the plot. The map image derived from National Platform for Common Geospatial Information Services (https://www.tianditu.gov.cn/), topographic map from Geospatial Data Cloud (http://www.gscloud.cn).

**Figure 2:**
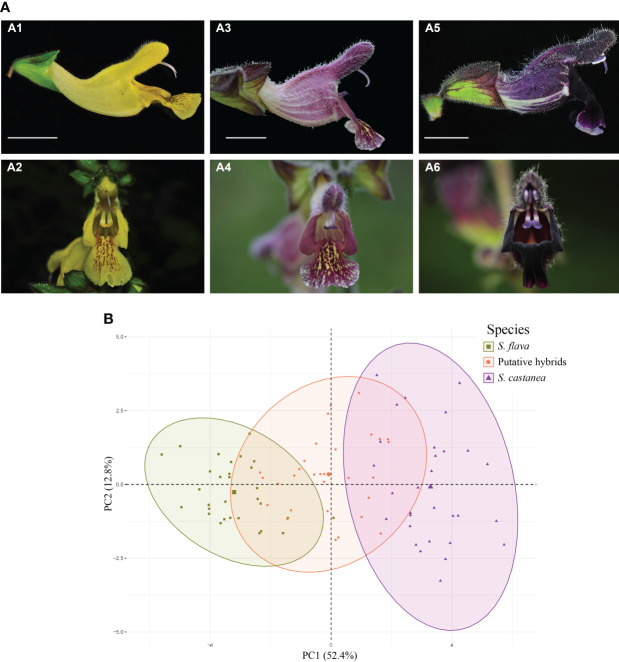
**(A)** Morphological differences between *Salvia flava* (A1–A2), *S*. *castanea* (A5–A6) and putative hybrid individuals (A3–A4), and **(B)** PCA plot of first two components based on data from the 16 quantitative morphological characters.

During field investigations, we found many individuals with forms that were morphologically intermediate between *S. flava* and *S. castanea* at two sympatric locations. These two locations were located in Mingyin (MY) village (27°25′6″N, 100°22′24″E) and Heishuihe (HSH) village (27°9′20″N, 100°15′34″E), in Yulong County, Lijiang City, SW China (**Figure 1A**). Parental species and putative hybrid individuals can be easily distinguished by morphology: *S. flava* has a bright yellow corolla and a hastate leaf blade; *S. castanea* has a purple corolla and an oblong-ovate leaf blade; the putative hybrids are intermediate in both flower color and leaf blade shape (**Figure 2A**; **Supplementary Figure 1**).

At the MY location, the majority of the individuals were *S. flava*, which is mainly distributed in areas with low light levels and high soil moisture. In contrary, only relatively few of the individuals were *S. castanea*, most of which could be found in the areas with sufficient light and relatively dry soil. The putative hybrids were fewest in number and were mainly distributed in the area where the two parental species overlapped (**Figure 1B**). In HSH, a road separated the populations of *S. flava* and *S. castanea*. The distance between the two populations was one kilometer. Due to the development of tourism, only relatively few individuals of the two species were present, and about 50 plants per species. The hybrids were mainly found near the *S. castanea* population. After careful investigation, only 7 hybrid plants were found. In 2019, 86 individuals (*S. flava*: 20; *S. castanea*: 29; putative hybrids: 37) from MY and 25 individuals (*S. flava*: 10; *S. castanea*: 10; putative hybrids: 5) from HSH were sampled. The leaves were dried and preserved in silica gel. Voucher specimens were deposited at the Herbarium of Shanghai Chenshan Botanical Garden (CSH; **Supplementary Table 1**). We declare that the acquisition of relevant materials and experiments mentioned below were in compliance with the law.

### 2.2 Measurement of morphological characters

We randomly selected a mature flower and a healthy basal leaf from each of 30 adult individuals from each taxon in MY for morphological measurements in August, 2019. A total of 16 morphological characters were measured: 1) pedicel length (PL); 2) calyx length (CL); 3) corolla length (COL); 4) corolla width (COW): 5) corolla height (COH); 6) length of upper lip of corolla (UL); 7) corolla tube length (COTL); 8) entrance height (EH); 9) entrance width (EW); 10) length of lower arm of stamen to lower wall of entrance (LA); 11) filament length (FL); 12) connective length (CONL); 13) pistil length (PIL); 14) basal leaf length (BLL); 15) basal leaf width (BLW); 16) petiole length of basal leaf (PLBL). The relevant data were measured using digital vernier calipers with an accuracy of 0.01 mm, and classification of traits types followed Rieseberg et al. (2003). The R package FactoMineR (Lê et al., 2008) and factoextra v 1.0.4 (Kassambara and Mundt, 2017) in R v 3.6.3 (R Core Team, 2018) were used for principal component analysis (PCA).

### 2.3 Population genetic analysis

#### 2.3.1 DNA extraction, ddRAD-seq and SNP identification

The modified CTAB method was used to extract high quality genomic DNA from dried young leaves (Doyle, 1991). A Qubit 3.0 fluorometer (Thermo Fisher Scientific, Waltham, MA, USA) was used to detect the concentration of DNA, and the qualified samples were normalized to 20 ng/μl. The samples were sent to Guangzhou Jierui BioScience Co. Ltd. (Guangzhou, China) for ddRAD-seq. Library preparation was conducted following Peterson et al. (2012). The total DNA was double digested with the restriction enzymes *EcoRI* (NEB) and *Msel* (NBE). A gel extraction kit (Omega Bio-Tek, Norcross, GA, USA) was used to screen out 300–500 bp of fragments, and sequencing was conducted on an Illumina HiSeq X Ten platform (Illumina Inc., San Diego, CA, USA) in PE150 mode (0.5 G each sample).

SNP calling and genotyping were performed using the STACKS v. 2.52 pipeline (Rochette et al., 2019). Firstly, raw data was filtered and demultiplexed using the *process_radtags* with the len_limit set to 140 bp to trim low-quality reads. Subsequently, *ustacks* was used to merge short-reads into loci on a maximum likelihood framework. The minimum depth of coverage required to create a stack was set to two (m = 2), and the maximum nucleotide mismatches allowed between two stacks was set to five (M = 5). A catalog was constructed using *cstacks* with the number of mismatches allowed between sample loci set to one (n = 1). Moreover, *sstacks* was used to match against the loci of each sample and the catalog to confirm alleles. Finally, the *populations* program in STACKS was used to filter the loci and divide individuals into three populations (*S. flava*, *S. castanea* and putative hybrids) at each location. The key parameters were as follows: –min-populations (p): 3, –min-samples-per-pop (r): 0.5, –min-maf: 0.05, –max-obs-het: 0.5. The first SNP was selected from each locus to obtain unlinked SNPs for further analyses.

#### 2.3.2 ddRAD-seq analysis

PCA was performed using PLINK v.1.9 (Purcell et al., 2007) and visualized using R v.3.6.3. A Bayesian-based analysis was then performed using STRUCTURE v. 2.3.4 (Pritchard et al., 2000) to clarify the population genetic structure. The tested *K* values were set to from one to five, with 10 replicates per *K*. The run was set for a burn-in of 100,000 steps followed by 100,000 iterations of the Markov Chain Monte Carlo algorithm (MCMC) iterations. The optimal value of *K* was chosen using the delta-*K* method implemented in STRUCTURE HARVESTER v. 0.6.94 (Earl and Vonholdt, 2012), and the web application “Pophelper” (Francis, 2017) was used to visualize the STRUCTURE results.

The software Newhybrids v. 1.1 was used to calculate the posterior probability of sampled individuals assigned to different hybrid categories based on the Bayesian clustering method (Anderson and Thompson, 2002). Because the number of loci was limited by this program, we selected loci with *F*_ST _= 1 between parent species as diagnostic loci. The filtered dataset then contained 80 SNPs from the MY location and 379 SNPs from the HSH location for the Newhybrids analysis. The program was run using a burn-in of 100,000 followed by 100,000 MCMC iterations.

### 2.4 Reproductive isolation barriers in the hybrid zone

The following linear formula was used to measure the prezygotic barriers that affect cooccurrence following Sobel and Chen (2014):


$$RI=1-\frac{S}{S+U}$$

where S refers to the sharing degree of factors between species and U represents the proportion unshared (e.g., space, florescence, and pollinator).

For postzygotic barriers, the following equation was used (Sobel and Chen, 2014):


$$RI=1-2\times \left(\frac{H}{H+C}\right)$$

where H and C mean heterospecific and conspecific mating, respectively. The value of RI is the degree to which barrier in this stage impedes inter-specific gene flow and ranges from 0 (no barrier) to 1 (complete barrier).

#### 2.4.1 Geographic isolation

We evaluated the overlaps of geography and altitude in the distributions of *Salvia flava* and *S. castanea* after carefully checking the specimens of these species from 20 herbariums in China (**Supplementary Table 2**). In addition, we also examined records of field investigations data and online resources, including GBIF (https://www.gbif.org, accessed May 30, 2021), Global plants on JSTOR (https://plants.jstor.org, accessed May 30, 2021) and Chinese Virtual Herbarium (https://www.cvh.ac.cn, accessed May 30, 2021). Incomplete information and repeated collections of specimens were removed. To avoid the influence of geneflow caused by pollen and seed dispersal, we followed Cuevas et al. (2018), and defined the distance of any two records between *Salvia* species within 7km as sympatric.

#### 2.4.2 Phenological isolation

We observed and recorded the flowering period of our two study species in MY from July to October 2019. We recorded the date when the first flower opened and when the last flower withered in the hybrid zone, and in this way, we evaluated the degree of overlap in florescence of *S. flava* and *S. castanea*. At the same time, 15 plants from each species (one flower per individual) were randomly selected and sealed with gauze bags to record the anthesis of a single flower.

#### 2.4.3 Floral isolation

To explore the effect of the corolla reflectance spectrum of the parental species and the putative hybrids on the attraction of potential pollinators, we used a USB2000+ miniature fiber optic spectrometer with a DH-2000-BAL deuterium-halogen light source (Ocean Optics, Dunedin, FL, USA) to analysis the light reflection patterns at different wavelengths (Ma et al., 2016). In this study, 30 mature flowers were randomly selected from each taxon for measurement (one flower per plant). Because in these *Salvia* species, the lower lip of the corolla is important in pollinator attraction, we measured the position of the lower lip. The measurements ranged from 250 to 850 nm, in 0.45 nm increments.

Three healthy inflorescences from the three taxa (each from different individuals) were randomly selected, marked and sealed with parchment paper bags to isolate from pollinators. When most of flowers were in bloom, the inflorescences were cut off and the decayed or withered flowers were removed, leaving 30 flowers per inflorescence. The cut end of each inflorescence was wrapped in cotton balls soaked in 2% sucrose solution and was sealed using parafilm (Pechiney, French). To avoid contamination with other chemicals, samples were enclosed in Tedlar bags (Dupont, USA) and were brought to the laboratory for collection and analysis of floral volatile compounds.

The composition of volatile compounds in flowers was measured using headspace solid-phase micro-extraction combined with gas chromatography-mass spectrometry (HS-SPME-GC-MS; Chen et al., 2014, 2015). Samples were analyzed using an Agilent Technologies HP 6890 gas chromatograph (GC) equipped with an HP-5MS column (30 m × 0.25 mm inner diameter, 0.25 mm film thickness) and linked to an HP 5973 mass spectrometer (MS).

The SPME holder was fitted with a 65 μm polydimethylsiloxane/divinylbenzene fiber (Supelco, Bellefonte, PA, USA) and pre-desorption for 15 min and heating to 200°C. The fiber was then placed in the atmosphere around the samples, and GC-MS analysis was performed directly after adsorption for 50 minutes. The split inlet and MS were held at 250°C. High purity helium was used as the carrier gas, at a flow rate of 1ml/min. Column temperature started at 40°C (5 min. hold) and was programmed to rise to 280°C (20min. hold) at 3°C/min. The MS were taken at 70 eV (in EI mode) and samples were scanned from m/z 35–500. Compounds were preliminarily identified using the Wiley 7n.1 and NIST98.L mass spectral library, and the average relative amounts (%) were determined based on peak area measurements. PCA and graphing of the relative contents of compounds was completed in the R packages FactoMineR and factoextra.

#### 2.4.4 Pollinator-mediated reproductive isolation

In August 2019, we observed and recorded floral visitors to *S. flava* and *S. castanea* at MY. Preliminary observation suggested that there were no nocturnal pollinators, so we only recorded from 8:00–19:00 in the daytime. We assigned multiple people to observe and record characteristics of pollinators, e.g., species, duration time and visiting behaviors. Observations were carried out in two different ways. (1) On August 17, 19, 23 and 24 in 2019, we randomly selected four individuals per taxon and observed them continually for four discontinuous sunny days to examine the pollinator assemblages in three natural settings dominated by *S. flava*, *S. castanea* and hybrid plants, respectively. (2) Parental species were artificially arranged into two plots, one of which was located in the *S. flava*-dominated area (plot 1) and the other in the *S. castanea*-dominated part (plot 2). In each plot, six individuals of *S. flava* and six of *S. castanea* were planted alternating (**Supplementary Figure 2**). Observations in the artificial plot setting were conducted over a total of seven discontinuous days from 18^th^ – 26^th^ August in 2019. Observations were not carried out on August 20^th^ and August 22^ed^ due to rain. Plants in the artificial plots were arranged in a grid structure with a distance of 50 cm between each individual, following Ma et al. (2019). The number of bloom flowers of *S. flava*, *S. castanea* and the hybrids varied from 60 to 90 during pollination observation in the natural condition. While in each artificial plot, different species of plants were pretreated to the same number of flowers. The number of flowers of each species observed was about 100 in plot-1 and 190 in plot-2. The mature plants were transplanted to the corresponding positions in advance for careful maintenance, and pollination observation was carried out after they bloom normally.

To calculate the ethological reproductive isolation, the formula employed by Natalis and Wesselingh (2013), 1 – (No. cross-species foraging bouts/total number of foraging bouts) × (No. heterospecific transitions/all transitions) was applied in this study. One bout refers to the entire visit of a pollinator to the plot, from entering the plot to leaving, and transition refers to a pollinator moving from one plant to another within one bout.

Accordingly, following Bateman (1951) and Waser (1986), we calculated the Bateman’s constancy Index (BI) as:


$$\frac{{\left(aa\;\times \;bb\right)}^{\frac{1}{2}}\;–\;{\left(ab\;\times \;ba\right)}^{\frac{1}{2}}}{{\left(aa\;\times \;bb\right)}^{\frac{1}{2}}\;+\;{\left(ab\;\times \;ba\right)}^{\frac{1}{2}}}$$

where aa and bb are within-species transitions, while ab and ba are heterospecific transitions. The BI ranges from -1 (complete inconstancy) to +1 (complete constancy), and 0 means the pollinators forage randomly.

#### 2.4.5 Hand pollination experiments

In order to evaluate the post-zygotic isolation between parental species, we carried out a series of hand pollination experiments in August 2019 at MY. 35 flowers randomly selected from 25 plants (1–2 flowers per plant) were used for each pollination treatment. Pollen donors (between 20 and 23 individuals), growing at a distance of more than 10 m from maternal plants. In addition, geitonogamous and xenogamous pollination experiments were also performed to test the fertility of the hybrids, including 35 flowers per treatment from 20 randomly selected maternal individuals and 18 pollen donor plants. The steps of the pollination experiments were as follows. When the flowers were about to open, they were covered with gauze bags, and the anthers were removed before anthesis. If they were in full bloom, hand pollination treatments were carried out and the flowers were then covered again until 72 h before being exposed to avoid disturbance from natural pollinators. The seeds from each treatment were counted on September 25 to October 15 in 2019.

#### 2.4.6 Total isolation

The following formula was used to calculate total RI and absolute contribution (AC) of each barrier to total RI, following Ramsey et al. (2003):


$$RC_{n}=\frac{AC_{n}}{T}$$

In the above formula, T is total reproductive isolation and RC means the relative contribution at a certain stage of reproductive barrier to total RI, while n means the n^th^ reproductive barrier in the life history. Due to the fact that the RI of different stage in the two *Salvia* species may be asymmetric, we calculated RI separately for each species and considered the isolation of sympatric location.

### 2.5 Data analysis

Morphological traits and volatile compounds among the three taxa were analyzed using a one-way ANOVA. The significance of differences between the means was determined using standard F statistic, and the Bonferroni test was employed for post hoc pairwise comparisons. Where the data did not satisfy the criterion of homogeneity of variance, a Welch statistic was employed, and post hoc comparisons were performed using the Tamhane’s test (Liao et al., 2021). In addition, single flower anthesis, preference of pollinators, fruit and seed sets between *S. flava* and *S. castanea* were assessed using a non-parametric Mann-Whitney U-test. For the hand cross-pollination experiments, two factors may influence the fruit and seed set, i.e., mother species and cross type (intraspecific or interspecific). We therefore used a two-factor ANOVA to test the effects of different factors on fruit and seed production. All of these tests were carried out in SPSS 16.0 for Windows (Chicago, IL, USA).

## 3 Results

### 3.1 Morphological analyses

The PCA based on data of 16 morphological characters was able to distinguish *Salvia flava* and *S. castanea* well through PC1 (variance explained = 52.4%) and PC2 (variance explained = 12.8%). Moreover, the putative hybrid individuals clustered into one group, which was intermediate between the *S. flava* and *S. castanea* clusters (**Figure 2B**). Eight of the 16 morphological traits studied were intermediate in the putative hybrids. Intriguingly, we also found that the length of the connective in hybrid plants was significantly shorter than in either parental species (**Supplementary Table 3**).

### 3.2 Population genetic results

#### 3.2.1 ddRAD sequencing

After quality filters had been applied, a total of 944,878,058 reads were obtained, with each sample yielding an average of 7.8 Million reads. Mean locus coverage for each sample was 61.72 ×, ranging from 6.8 × to 141.33 ×. A catalog of 2,741,910 putative loci was constructed in *cstacks*, and through the *populations* program in STACKS, 2,216 SNPs and 3,895 SNPs were retained from the MY and HSH locations, respectively (**Supplementary Table 4**).

#### 3.2.2 Population genetic structure

**Figure 3:**
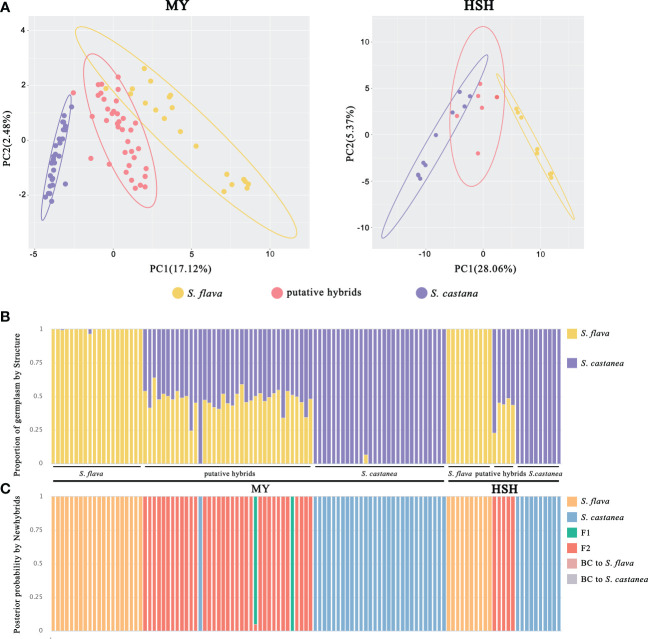
Population genetic analyses of *S*. *flava*, *S*. *casatanea* and the putative hybrids based on SNP variation. **(A)** PCA plot of the first two components for MY and HSH locations. The ellipses represent the 95% confidence interval. **(B)** The STRUCTURE plot with *K* = 2. **(C)** Genotype class assignment by Newhybrids based on diagnostic loci.

The genetic differentiation coefficients *F*_ST_ between *S. flava* and *S. castanea* were the highest in the two hybrid zones (**Supplementary Tables 5, 6**). A PCA based on SNPs was able to separate *S. flava* and *S. castanea* from the two hybrid zones along PC1 (variance explained = 17.12% in MY and 28.06% in HSH; **Figure 3A**).

STRUCTURE analysis based on all SNPs indicated that *K* = 2 was the optimal value of Δ*K* at both MY and HSH (**Supplementary Figure 3**), suggesting that all samples can be divided into two genetic clusters corresponding to *S. flava* and *S. castanea*. With the exception of one individual (MYH19) in MY that was mainly assigned to *S. castanea*, all the remaining putative hybrids showed a mixture of genetic components from both their parental species (**Figure 3B**).

The output from Newhybrids based on diagnostic sites was consistent with the clustering pattern seen in the STRUCTURE analysis. For both hybrid zones, the Newhybrids assignment of parental individuals corresponded to the morphological assessment, and these individuals had a posterior probability to nearly 100%. In addition, MYH19 was also identified as pure *S. castanea* with posterior probabilities > 90% in Newhybrids. Interestingly, almost all of the remaining hybrid individuals at both of the two locations were assigned to the F_2_ class, with the exception of two individuals were predicted to be F_1_s at MY (**Figure 3C**).

### 3.3 Reproductive isolation

#### 3.3.1 Geographic isolation examine

Through examination of specimens, information of field investigations and online data, we finally obtained a total of 103 pieces of distribution information (*Salvia flava*: 36; *S. castanea*: 58; **Figure 1A**). The geographic isolation of *S. flava* was calculated to be 0.83 and *S. castanea* was 0.88. Moreover, according to 231 pieces of elevation information (*S. castanea*: 113; *S. flava*: 118), *S. castanea* grows at an average altitude of 3128 m (range 1300–4200 m), which is significantly higher than that of *S. flava* (2000m, range 2700–4434 m; Z = - 4.81, P< 0.001).

#### 3.3.2 Phenological isolation

There were no significant differences in single flower anthesis between *S. flava* and *S. castanea* (3.87 ± 0.35 d vs. 3.67 ± 0.41 d; n = 15; Z=–0.482, P= 0.63). However, we found a large flowering period overlap between the two species, where *S. flava* flowered from July 20 to September 25, and *S. castanea* from August 1 to October 15. The overlap in the flowering period was therefore 56 days, and the RI_phenology_ of *S. flava* and *S. castanea* was calculated to be 0.13 and 0.26, respectively.

#### 3.3.3 Floral isolation

The reflectance spectra of the lip of corolla are different in *Salvia flava* and *S. castanea*. The former has a clear peak at about 520nm and a slight peak at ca. 450nm. However, *S. castanea* only has a very insignificant peak at 450 nm, and no peak at 520 nm was detected. Similar to *S. flava*, hybrids have a peak at 450 nm and 520 nm, with the reflectance of the peak at 450 nm being close to that of *S. flava*, while the reflectance at 520 nm was significantly lower than that of *S. flava* (**Supplementary Figure 4A**).

A total of 15 and 16 compounds omitted from flowers of *S. flava* and *S. castanea*, respectively were identified (80.33% and 80.53% of total extracted mass, respectively). These volatile compounds could be divided into monoterpenes, sesquiterpenes, aromatics and aliphatic compounds, of which monoterpenoids represented the most extracted compounds in both species (**Supplementary Table 7**). Five compounds, representing 9.83% of the total, were extracted from the flowers of *S. flava* and were not present in *S. castanea*, while six compounds in *S. castanea* (30.02%) were not detected in *S. flava*. Most of the unique compounds extracted from the flowers of the two species were monoterpenes. We did not detect either m-cymene or borneol in the hybrids, although these compounds occurred in both parental species. In addition, ocimene was only found in hybrids, but only accounts for 0.56%.

PCA revealed that *S. flava* and *S. castanea* can be well separated along PC1 (variance explained = 62.5%) and that the hybrids are located in the middle coefficient position between the two parental species (**Supplementary Figure 4B**). The hybrids showed further differentiation from the parental species along PC2 (variance explained = 34%).

#### 3.3.4 Pollinator mediated isolation

**Figure 4:**
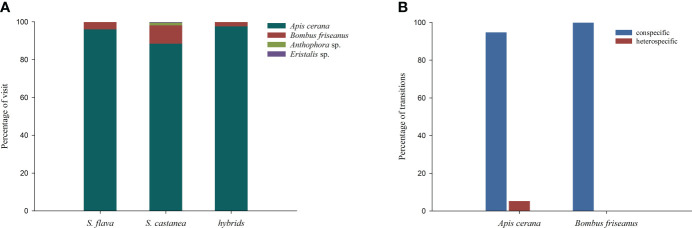
Proportion of flower visitors to *S*. *flava*, *S*. *castanea* and hybrid plants in a natural setting **(A)**, and transition percentages of pollinators in the manual plots **(B)**. Transition types were categorized as: conspecific (*S. flava*–*S. flava*, or *S*. *castanea*–*S*. *castanea*) and heterospecific (*S*. *flava–S*.*castanea*, or *S*. *castanea*–*S*. *flava*).

Observations of pollinators in the natural setting showed there were four species of effective pollinators (**Figure 4A**), *Apis cerana* (eastern hive bee), *Bombus friseanus* (bumblebee), *Anthophora* sp. and *Eristalis* sp. There were 176 observed visits to *S. flava* (169 visits from *A. cerana*; 7 visits from *B. friseanus*) and 219 obsserved visits to *S. castanea* (194 visits from *A. cerana*; 21 visits from *B. friseanus*; three visits from *An*. sp. and only one visit from *E.* sp.). Honeybees and bumblebees were therefore the most important pollinators shared by *S. flava* and *S. castanea*. According to the formula, RI_pollinator assemblage_ was zero for *S. flava* and 0.0183 for *S. castanea*. We also recorded the geitonogamous tendencies of the observed pollinators. Whether parents or hybrids, more than 50% of pollinators foraged multiple flowers per plant, and the average number of flowers per plant of *S. flava*, *S. castanea* and hybrids were 3.04 ± 2.63, 2.00 ± 1.35 and 2.41 ± 1.90, respectively.

A total of 52 pollination bouts were recorded in plot-1, including four interspecific bouts and 48 intraspecific bouts. Of the 70 transitions recorded, five were conspecific transitions and 65 were heterospecific transitions. In plot-2, however, a total of 129 bouts were observed, of which eight were heterospecific and 121 were conspecific. Furthermore, nine cross-specific transitions and 194 conspecific transitions were recorded. Thus, we estimated the RI from the foraging behavior of the pollinators as 0.9945 for *S. flava* and 0.9973 for *S. castanea*.

All the observed transitions mentioned above were seen in either *A. cerana* or *B. friseanus*. *B. friseanus* transitions occurred only within taxa, while *A. cerana* transitions were of all types. The honeybee made mostly conspecific transitions, which accounted for 94.78% all transitions (**Figure 4B**). BI in plot-1 was calculated as 0.8447 and in plot-2 was calculated as 0.8411, i.e., pollinators had high flower constancy in any given foraging bout. Interestingly, pollinators were highly attracted to the dominant plant species in particular plots. For instance, in plot-1, located in the *S. flava*-dominated area, pollinators showed great interest in visiting *S. flava*, and the visit preference of *S. flava* was significantly higher than that of *S. castanea* (0.7681 vs. 0.2319; Z = – 8.393, P< 0.001). In contrast, pollinators were more likely to forage *S. castanea* in plot-2 (in the area dominated by *S. castanea*), and showed a significantly lower preference for *S. flava* than for *S. castanea* (0.1666 vs. 0.8334; Z = – 8.393, P< 0.001).

#### 3.3.5 Post-zygotic isolation

**Figure 5:**
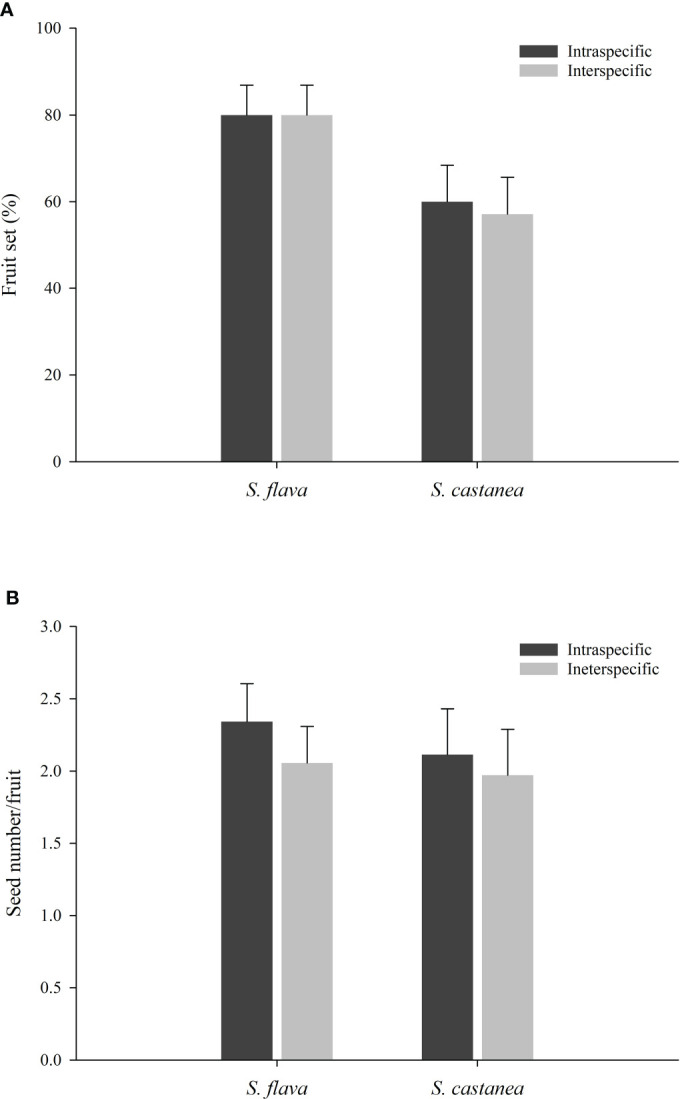
Fruit set **(A)** and seed number per fruits **(B)** from inter- and intra-specific pollinations of *S*. *flava* and *S*. *castanea* (± S.E.).

The results of our hand pollination experiments showed that higher fruit set was obtained when *S. flava* was the mother species (F = 7.759, P = 0.006), whereas the type of cross (i.e., intraspecific and interspecific) had no effect on fruit set. Furthermore, neither mother species nor cross type caused significant differences in seed set (**Supplementary Tables 8, 9**). According to the results from the fruit set experiments, (**Figure 5**; **Supplementary Table 8**), the RI _fruit set_ for *S. flava* was calculated to be 0 whereas the RI _fruit set_ of *S. castanea* was 0.0244. Similarly, the RI by seed set was calculated to be 0.0649 for *S. flava* and 0.0350 for *S. castanea*. In addition, although the fruiting and seed set were lower in hybrid plants than in either parent species, the hybrids still showed some degree of fertility (**Supplementary Table 8**). There was no significant difference in fruit set following geitonogamous or xenogamous pollinations in hybrids (48.57% vs. 42.86%; both n = 35; Z = –0.476, P= 0.634), and the seed number per fruit was similar in both cases (0.9429 vs. 1.0286; both n = 35; Z = –0.187, P= 0.851).

## 4 Discussion

This is the first report of natural hybridization of the East Asian *Salvia* clade (Subgenus *Glutinaria*) that combines both phenotypic and molecular evidence. In this study, most morphological characters seen in the hybrid plants were either intermediate between those of the parents or resembled one or other of the parental species, implying that these were early generation hybrids (Rieseberg and Carney, 1998). However, we detected transgressive characters, suggesting the occurrence of genome recombination even in some of these early generation hybrids (Rieseberg et al., 2003; Abbott et al., 2013). Although a hybrid individual was found to be genetically similar to *S. castanea*, however, the results from our STRUCTURE analysis shower that the remaining hybrid individuals displayed genetic admixture between the two parents (**Figure 3**), and these data support the morphological evidence.

In general, there are two prerequisites for occurrence of natural hybridization. On one hand, there should be the chance of interspecific pollination and the production of at least a few seeds between two parents. For *S. flava* and *S. castanea*, their sympatric distribution, overlapping flowering periods and shared pollinators (bees) would allow interspecific pollination in the prezygotic reproductive stage, and furthermore, F_1_ seeds can be produced by hand interspecific pollination in the postzygotic stage. On the other hand, seeds from interspecific pollination must be able to germinate and the seedlings survive to flowering (In this study, quantitative assessment was not carried out because very few mature seeds remained for harvest due to storms when the fruits were about to ripen). This second prerequisite is often associated with habitat disturbance, which can not only break ecological isolation to a certain extent, but can also create intermediate habitats and promote for hybrids survival (Anderson, 1948; Arnold, 1997; Rieseberg and Carney, 1998). For this study, tourism, reclamation, road building and grazing were recorded in both hybrid zones, these may lead to creation of suitable habitats for survival of these natural hybrids.

Although hybridization indeed occurred between these two *Salvia* species, strong reproductive isolation remained between *S. flava* and *S. castanea* due to geographic isolation at the species level and to pollinator mediated isolation in sympatric populations (**Table 1**). Our results support the hypothesis that for recently diverged species, prezygotic barriers play a much important role than does postzygotic RI (Ramsey et al., 2003; Lowe and Abbott, 2004; Kay, 2006; Natalis and Wesselingh, 2013; Ma et al., 2016, 2019). In *S. flava* and *S. castanea*, higher fruit set (80% and 57.14%, respectively) was obtained when flowers received interspecific pollination. Intriguingly, context-dependent behavior of the shared pollinators, calculated by constancy index (0.8447 in plot-1 and 0.8411 in plot-2), largely impeded inter-specific pollen movement, which suggesting that although the flowers of these two species are not widely divergent, pollinators still distinguish between them. This means that the distribution pattern itself can form RI, which is particularly in common scenarios where there is a cluster of individuals growing within a different species in natural population (Natalis and Wesselingh, 2013; Ma et al., 2019). The context-dependent foraging behavior might reflect pollinator learning habits, for example, pollinators always forage in areas (even very small size) with flowering individuals of the same plant species. We can further speculate that given hybridization was found to have occurred in different sympatric areas for the two parental species, RI can be formed and be largely determined by the proportion of flowering individuals of each parental species growing in each area, without the obvious divergence of flora characters that has been believed to be key for pollinator mediated RI.

**Table 1:** Contribution of assessed barriers to reproductive isolation (RI) between *S*. *flava* and *S*. *castanea*.

Isolation barrier	Components of RI		Absolute contributions to total RI		Absolute contributions to total RI (In Sympatry)	
	*S*. *flava*	*S*. *castanea*	*S*. *flava*	*S*. *castanea*	*S*. *flava*	*S*. *castanea*
Prezygotic						
Geographic	0.81	0.88	0.81	0.88		
Phenology	0.13	0.26	0.0247	0.0312	0.13	0.26
Pollinator assemblage	0	0.0183	0	0.0016	0	0.0135
Pollinator ethological	0.9945	0.9973	0.1644	0.0869	0.8654	0.7245
Total			0.9991	0.9997	0.9954	0.9980
Postzygotic						
Fruit set	0	0.0244	0	0.0244	0	0.0244
Seed production	0.0649	0.0350	0.0649	0.0341	0.0649	0.0341
Total			0.0649	0.0585	0.0649	0.0585

One interesting finding is that most hybrids are probably F_2_s, which was previously believed to be rare (but see Ma et al., 2010), due to the fact that once F_1_s were produced, there were generally surrounded by large proportions of parental plants, and there was therefore a higher possibility of backcrossing between the F_1_s and their parents than of self-pollination within the F_1_s. Moreover, even a bit seeds of F_2_s was produced, lethal effects (as described in the Dobzhansky-Muller model of hybrid incompatibility; Dobzhansky, 1936; Muller, 1942) would act to remove some of the recombinant genotypes, but this is likely to be less of an issue for backcrossed genotypes. Therefore, two common genotypes for hybrids reported before were 1) F_1_- dominated (like *Rhododendron*, Milne et al., 2003; Zha et al., 2010; *Buddleja*, Liao et al., 2015, 2021; and *Ligularia*, Zhang et al., 2018; Hu et al., 2021a, 2021b), or 2) backcross-dominated (like *Quercus*, Lepais et al., 2009; Iris, Arnold et al., 2010; and *Primula*, Ma et al., 2014, 2019).

Unlike the common pattern of hybrid zones dominated by F_1_s or backcrosses, the F_2_-dominated hybrid zone reflect another evolutionary significance for hybrid speciation. The successful production of F_2_s, particular for the present study case, i.e., where a hybrid zone is dominated by F_2_s, suggesting that some degree of RI already exist between the hybrids and the parent species. As we discussed above, if F_2_s were produced from selfed F_1_s and other hybrids such as backcrosses were absent, further intermediate genotypes, like F_3_, or F_4_……would also be predicted. This would be accompanied by a recombination process by which the genome of the hybrid plants would be homologized, which is considered to be the key stage in homoploid hybrid speciation (HHS; Rieseberg, 1997; Mallet, 2007; Soltis and Soltis, 2009; Abbott et al., 2013).

Facilitated by our field investigation data, two clues, involving pollinator behavior and self-compatibility towards hybrids, can explain the unusual F_2_-dominated hybrid zone. Firstly, bees and/or bumblebees acting as the main pollinators of *Salvia* species are likely to be facilitating self-pollination (geitonogamy) between flowers of an F_1_ individual, as these pollinators often minimize inter-flower travel by preferentially foraging from adjacent flowers (Stout, 2007; Ma et al., 2015). Additionally, we evaluated the self-compatibility of hybrids in a hand self-pollination experiments, resulting in 48.57% fruit set rate and an average of 0.9429 seeds per fruit. This implies that there is potential for the continuous production of these intermediate genotypes of hybrids (50% genetic background per parent, see STRUCTURE results; **Figure 3**). Thus, we may be witnessing the early stages of HHS in *Salvia* providing that more intermediate hybrid genotypes of latter generations be produced and that reproductive barriers remaining strong enough to impede the formation of backcrossed hybrids. Hybridization is probably an important source of diversification in this genus on the QTP.

## Data availability statement

The Raw ddRAD-seq reads have been released and are public available in the National Center for Biotechnology Information (NCBI) database (can be viewed at https://www.ncbi.nlm.nih.gov/bioproject/PRJNA841072).

## Author contributions

YM and YW conceived the project and designed the experiments. YC, SZ, HX, DL, and YH performed field investigations and experiments. YC and YM analyzed and interpreted the data. YC drafted the manuscript. YC, YW, and YM revised the manuscript. All authors contributed to the article and approved the submitted version.

## Funding

This study was supported by the Specific Project for Strategic Biological Resources and Technology Supporting System from the Chinese Academy of Sciences (Grant No. ZSZY-001), the Science and Technology Program of Shanghai Science and Technology Committee (Grant No. 20392000600), the Ten Thousand Talent Program of Yunnan Province (Grant No. YNWR-QNBJ-2018-174) and the Chenshan Special Foundations from Shanghai Municipal Administration of Forestation and City Appearances (Grant No. G222402).

## Acknowledgments

We are grateful to Mr. Gang Yao and Mr. Yubing Zhou for help with data analysis, Ms. Songting Du, and Mr. Zhi Chen for providing photos and Dr. Gao Chen for help in the collection of floral volatile compounds. We also thank Mr. Fengmao Yang and Ms. Yiqing Wang for their constructive comments on the manuscript.

## Conflict of interest

The authors declare that the research was conducted in the absence of any commercial or financial relationships that could be construed as a potential conflict of interest.

## Publisher’s note

All claims expressed in this article are solely those of the authors and do not necessarily represent those of their affiliated organizations, or those of the publisher, the editors and the reviewers. Any product that may be evaluated in this article, or claim that may be made by its manufacturer, is not guaranteed or endorsed by the publisher.
