# Supplementary material for: Unusual patterns of hybridization involving two alpine Salvia species: Absence of both F1 and backcrossed hybrids
Source: Front Plant Sci. 2022 Oct 18;13:1010577. doi: 10.3389/fpls.2022.1010577 (PMC9623266; doi:10.3389/fpls.2022.1010577)
Supplement: Supplementary file 1 [file DataSheet_1.docx]

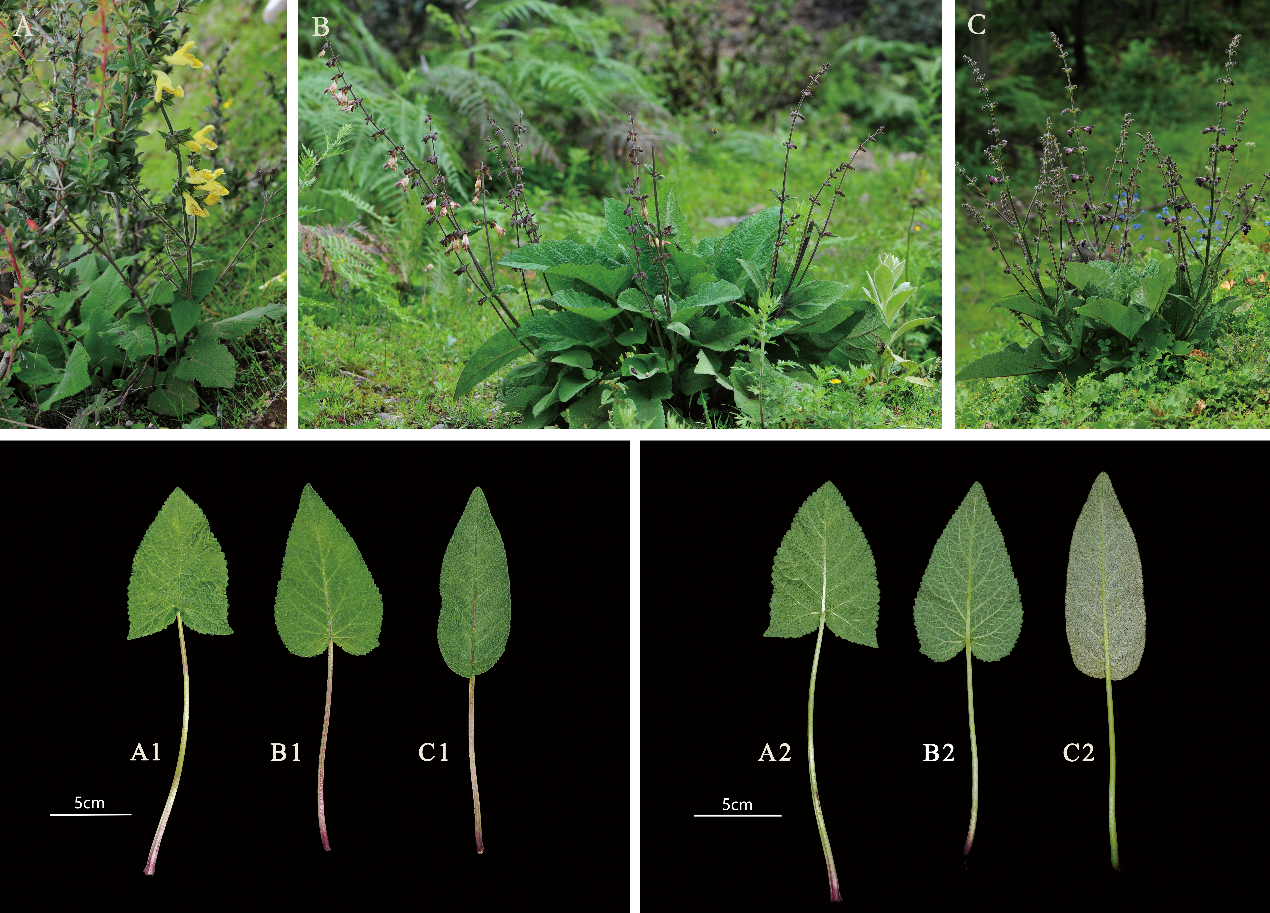


**Supplementary Figure 1.** Differences of habit and leaf blade shape between *S*. *flava* (A–A2), *S*. *castanea* (C–C2) and the putative hybrids (B–B2)


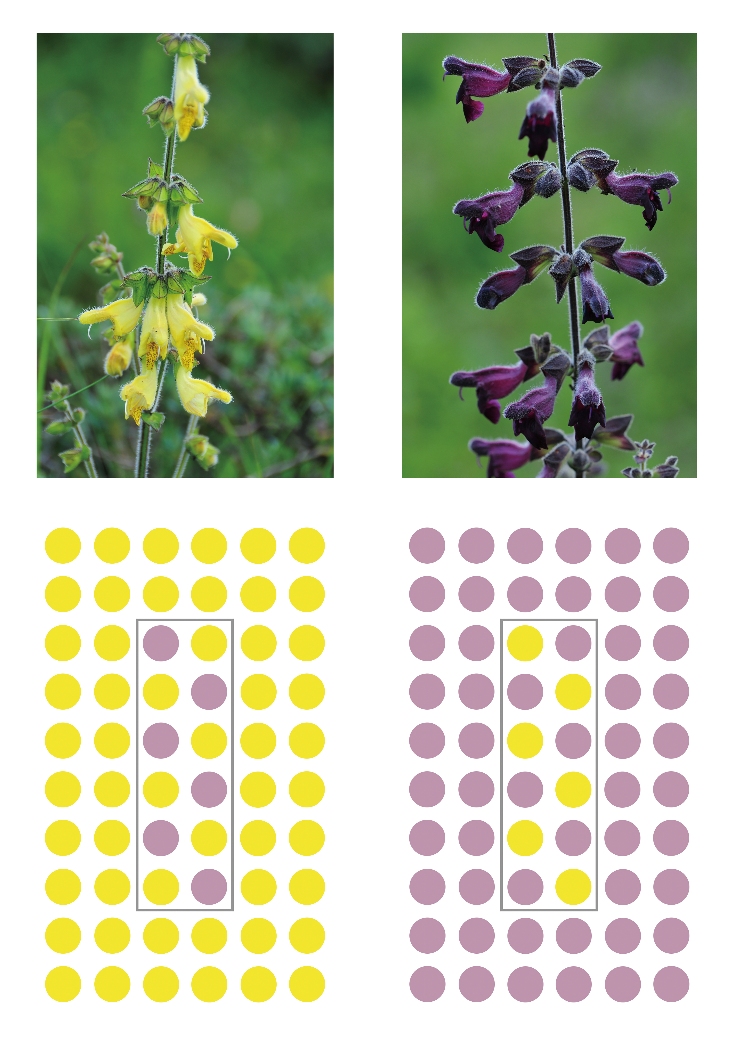


**Supplementary Figure 2.** Pollinator transition observations of arranged species in *S*. *flava* and *S*. *castanea* dominant plots


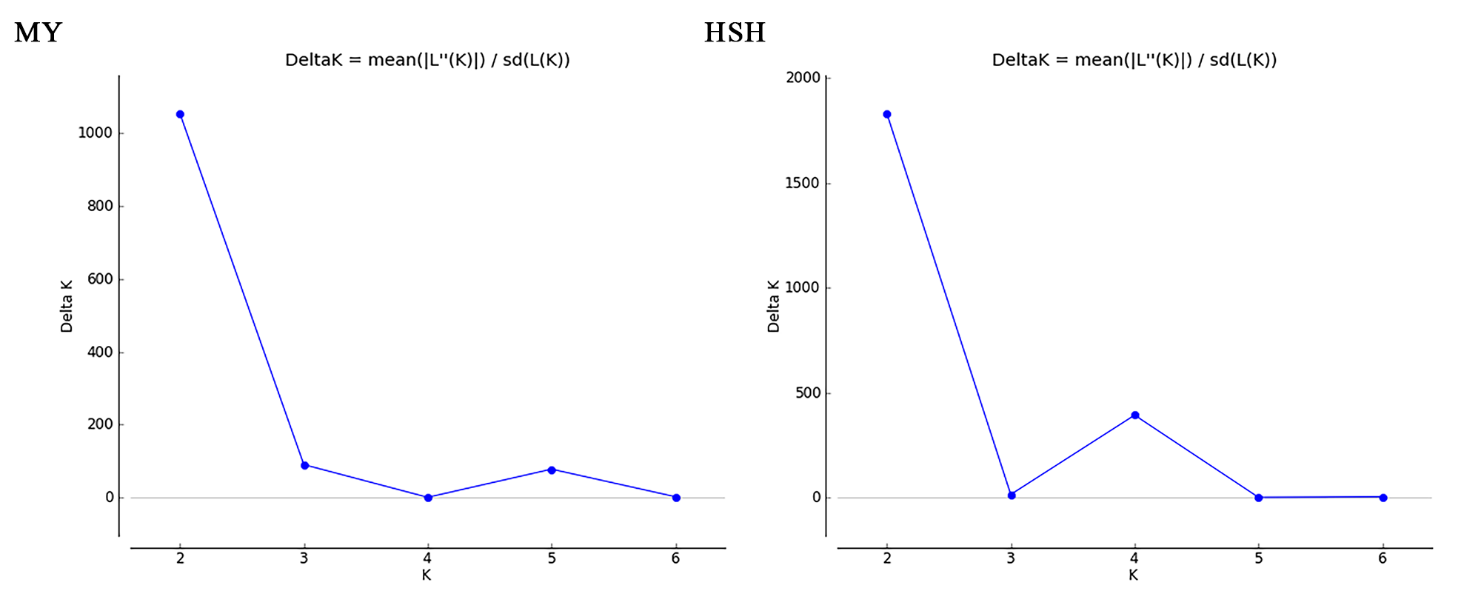


**Supplementary Figure 3.** Rate of change in the log probability of data between successive *K* values of MY and HSH


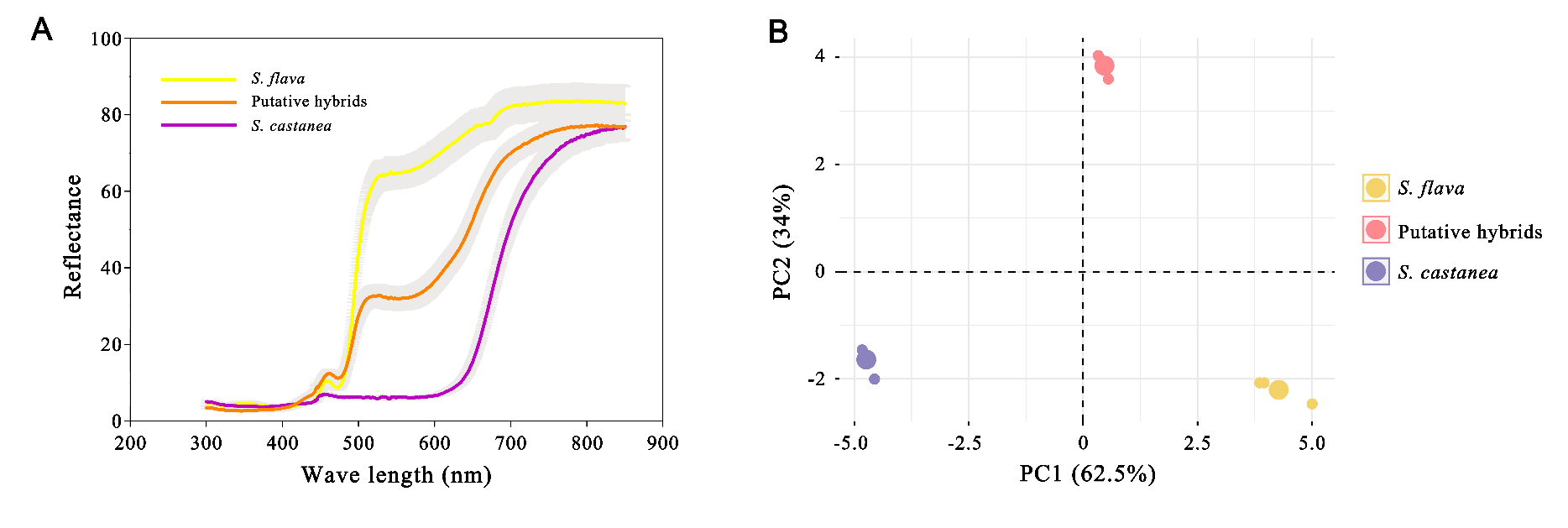


**Supplementary Figure 4.** Reflectance spectrum of the lower lip of corolla (A) and PCA plot of the first two components extracted for *S*. *flava*, *S*. *castanea* and the putative hybrids (B)

**Supplementary Table 1.** Information of specimens of the *Salvia* species in this study

| Species | Locality information | Locations | Coll. No. | Collectors | Herbarium | Longitude (E) | Latitude (N) | Altitude (m) |
| --- | --- | --- | --- | --- | --- | --- | --- | --- |
| *S*. *flava* | Yulong, Lijiang, Yunnan, China | MY | S1362 | Yukun Wei, Hanwen Xiao and Yuhang Chang | CSH | 100°22'20" | 27°25'06" | 3300 |
| Putative hybrids |  |  | S1361 |  |  | 100°22'24" | 27°25'06" | 3268 |
| *S*. *castanea* |  |  | S1360 |  |  | 100°22'24" | 27°25'07" | 3268 |
| *S*. *flava* |  | HSH | S1134 | Yukun Wei, Yanbo Huang and Gang Yao |  | 100°15'41" | 27°09'14" | 3200 |
| Putative hybrids |  |  | S0403 | Qi Wang Yanbo Huang and Jianjun Sun |  | 100°15'34" | 27°09'20" | 3045 |
| *S*. *castanea* |  |  | S1133 | Yukun Wei, Yanbo Huang and Gang Yao |  | 100°15'41" | 27°09'14" | 3026 |

**Supplementary Table 2.** The code of herbarium of specimens examined

| Herbarium | Herbarium |
| --- | --- |
| BNU | IMD |
| CCNU | KUN |
| CDBI | LBG |
| CMMI | NAS |
| CSH | PE |
| HIB | SHM |
| HITBC | SHMI |
| IBK | SZ |
| IBSC | WH |
| IMC | WUK |

**Supplementary Table 3.** Differences in morphological characters between *S*. *flava*, putative hybrids and *S*. *castanea* (n=30) at the MY location

| Characters (mm) | Mean ± S.D | | | H | F | P value |
| --- | --- | --- | --- | --- | --- | --- |
|  | *S*. *flava* | Putative hybrids | *S*. *castanea* |  |  |  |
| PL | 4.37 ± 0.82^a^ | 5.32 ± 1.46^b^ | 6.18 ± 1.62^b^ | 22.253 |  | **< 0.001** |
| CL | 10.55 ± 1.74^a^ | 13.72 ± 1.26^b^ | 15.49 ± 1.90^c^ | 59.836 |  | **< 0.001** |
| COL | 27.52 ± 2.90^a^ | 33.83 ± 2.98^b^ | 41.78 ± 3.03^c^ |  | 173.59 | **< 0.001** |
| COW | 10.84 ± 1.64^a^ | 12.01 ± 2.19^a^ | 14.06 ± 2.39^b^ |  | 18.115 | **< 0.001** |
| COH | 17.07 ± 2.91^a^ | 20.57 ± 2.10^b^ | 22.49 ± 3.17^b^ | 40.619 |  | **< 0.001** |
| UL | 8.94 ± 1.29^a^ | 9.71 ± 1.14^b^ | 10.44 ± 1.21^b^ |  | 11.451 | **< 0.001** |
| COTL | 20.42 ± 2.47^a^ | 25.91 ± 2.43^b^ | 32.11 ± 2.32^c^ |  | 176.823 | **< 0.001** |
| EH | 6.01 ± 0.89^a^ | 6.91 ± 0.81^b^ | 7.85 ± 1.06^c^ |  | 29.914 | **< 0.001** |
| EW | 4.93 ± 0.72^a^ | 5.72 ± 0.73^b^ | 7.51 ± 0.87^c^ | 58.278 |  | **< 0.001** |
| LA | 2.10 ± 0.69^a^ | 3.14 ± 0.79^b^ | 4.04 ± 0.81^c^ |  | 48.655 | **< 0.001** |
| FL | 6.44 ± 0.62a | 7.31 ± 0.67^b^ | 9.10 ± 0.94^c^ |  | 95.423 | **< 0.001** |
| CONL | 9.07 ± 1.03^a^ | 8.44 ± 0.87^b^ | 9.09 ± 1.12^a^ |  | 4.089 | **0.020** |
| PIL | 29.88 ± 2.67^a^ | 34.67 ± 2.56^b^ | 41.08 ± 2.96^c^ |  | 126.855 | **< 0.001** |
| BLL | 78.59 ± 17.48^a^ | 148.23 ± 43.41^b^ | 186.08 ± 53.75^b^ | 57.534 |  | **< 0.001** |
| BLW | 57.46 ± 14.53^a^ | 81.51 ± 22.30^b^ | 93.52 ± 30.13^b^ | 29.290 |  | **< 0.001** |
| PLBL | 99.51 ± 30.31^a^ | 113.98 ± 37.78^a^ | 106.32 ± 44.45^a^ |  | 1.091 | 0.34 |

*PL* pedicel length, CL calyx length, *COL* corolla length, *COW* corolla width, *COH* corolla height, *UL* length of upper lip of corolla, *COTL* corolla tube length, *EH* entrance height, *EW* entrance width, *LA* length of lower arm of stamen to lower wall of entrance, *FL* filament length, *CONL* connective length, *PIL* pistil length, *BLL* basal leaf length, *BLW* basal leaf width, *PLBL* petiole length of basal leaf

**Supplementary Table 4.** Some results of the RAD-seq library obtained in this study

| **Species** | **Sample ID** | **Total** | **No Rad Tag** | **Low Quality** | **Retained** | **Locus Coverage** | **sd.** | **Locus** |
| --- | --- | --- | --- | --- | --- | --- | --- | --- |
| *S*. *flava* | HSHF1 | 25754612 | 121952 | 776 | 25631884 | 130.58 | 200.91 | 3599122(14.1%) |
|  | HSHF7 | 16602910 | 270984 | 1022 | 16330904 | 57.19 | 65.26 | 6495750(40.4%) |
|  | HSHF9 | 914878 | 72357 | 50 | 842471 | 9.7 | 19.41 | 2552734(42.4%) |
|  | HSHF13 | 4281922 | 153708 | 241 | 4127973 | 57.42 | 59.54 | 1993339(50.1%) |
|  | HSHF15 | 947640 | 95058 | 36 | 852546 | 6.87 | 18.6 | 559347(31.5%) |
|  | HSHF17 | 7451144 | 185158 | 476 | 7265510 | 70.76 | 73.25 | 3196717(45.0%) |
|  | HSHF21 | 3078346 | 37119 | 77 | 3041150 | 12.06 | 29.32 | 4065019(33.3%) |
|  | HSHF23 | 2982198 | 34735 | 94 | 2947369 | 9.27 | 27.66 | 2175465(29.5%) |
|  | HSHF25 | 12079392 | 250155 | 781 | 11828456 | 73.23 | 79.69 | 4992374(43.1%) |
|  | HSHF27 | 4898144 | 127488 | 327 | 4770329 | 66.95 | 68.22 | 2289662(49.2%) |
| *S*. *castanea* | HSHC3 | 3581308 | 89010 | 230 | 3492068 | 100.44 | 111.4 | 1512021(44.3%) |
|  | HSHC11 | 8182666 | 59953 | 6649 | 8116064 | 77.22 | 80.52 | 2933835(36.4%) |
|  | HSHC13 | 1349376 | 13188 | 1116 | 1335072 | 9.69 | 23.83 | 1446465(40.4%) |
|  | HSHC15 | 1642404 | 13456 | 1304 | 1627644 | 9.09 | 24.44 | 1560518(35.7%) |
|  | HSHC17 | 1143248 | 19109 | 883 | 1123256 | 8.33 | 23.47 | 1506282(35.6%) |
|  | HSHC19 | 26328396 | 132012 | 21213 | 26175171 | 77.43 | 96.85 | 8143217(31.3%) |
|  | HSHC21 | 11776510 | 310377 | 764 | 11465369 | 62.61 | 68.84 | 4860118(43.1%) |
|  | HSHC25 | 21428214 | 267531 | 1668 | 21159015 | 61.73 | 71.22 | 8036356(38.5%) |
|  | HSHC27 | 3819146 | 82658 | 249 | 3736239 | 51.3 | 55.28 | 1779736(48.7%) |
|  | HSHC29 | 7232580 | 65263 | 5694 | 7161623 | 79.05 | 84.44 | 2566941(36.1%) |
| putative hybrids | HSHH1 | 25172350 | 119370 | 20393 | 25032587 | 78.51 | 90.51 | 8614348(34.6%) |
|  | HSHH4 | 4540764 | 24426 | 3611 | 4512727 | 24.35 | 51.84 | 1704945(32.9%) |
|  | HSHH5 | 5626174 | 37796 | 4428 | 5583950 | 48.06 | 63.13 | 2037487(36.1%) |
|  | HSHH6 | 13654036 | 78220 | 10897 | 13564919 | 63.41 | 80.31 | 4396864(32.6%) |
|  | HSHH7 | 9440420 | 52791 | 7591 | 9380038 | 60.63 | 73.92 | 3160854(33.9%) |
| *S*. *flava* | MYF1 | 6978152 | 36439 | 4234 | 6937479 | 122.44 | 119.2 | 2823602(40.9%) |
|  | MYF5 | 2630218 | 24280 | 1618 | 2604320 | 12.53 | 34.45 | 2334444(38.1%) |
|  | MYF6 | 5624124 | 33616 | 3493 | 5587015 | 125.18 | 120.64 | 2295963(41.4%) |
|  | MYF7 | 1250252 | 10864 | 811 | 1238577 | 10.24 | 27.08 | 1763459(39.5%) |
|  | MYF9 | 8321728 | 48927 | 5103 | 8267698 | 127.44 | 124.66 | 3199643(39.0%) |
|  | MYF10 | 6831670 | 41361 | 4269 | 6786040 | 128.46 | 123.25 | 2689507(39.9%) |
|  | MYF12 | 13324450 | 69782 | 8341 | 13246327 | 125.92 | 126.38 | 4704303(35.7%) |
|  | MYF13 | 3406946 | 27936 | 2097 | 3376913 | 125.98 | 119.39 | 1465695(43.8%) |
|  | MYF14 | 742116 | 43523 | 30 | 698563 | 19.66 | 49.13 | 304788(37.8%) |
|  | MYF15 | 5662828 | 38534 | 3423 | 5620871 | 127.6 | 122.33 | 2320886(41.6%) |
|  | MYF17 | 37276070 | 227612 | 1057 | 37047401 | 116.69 | 177.25 | 5378205(14.6%) |
|  | MYF18 | 14092786 | 85431 | 412 | 14006943 | 121.71 | 178.39 | 2316067(16.6%) |
|  | MYF21 | 7220906 | 79287 | 192 | 7141427 | 31.37 | 83.56 | 1261049(17.1%) |
|  | MYF22 | 2968112 | 51572 | 212 | 2916328 | 15.78 | 46.19 | 1410839(35.6%) |
|  | MYF24 | 2849576 | 26007 | 81 | 2823488 | 9.54 | 35.86 | 945958(23.4%) |
|  | MYF25 | 24810838 | 338054 | 1617 | 24471167 | 25.29 | 64.86 | 8929355(28.5%) |
|  | MYF26 | 603644 | 31616 | 32 | 571996 | 6.8 | 23.3 | 633467(34.4%) |
|  | MYF27 | 4826714 | 38201 | 130 | 4788383 | 13.52 | 53.78 | 1224714(21.4%) |
|  | MYF28 | 19082766 | 79088 | 540 | 19003138 | 116.44 | 174.65 | 2817897(14.9%) |
|  | MYF31 | 1400062 | 17114 | 27 | 1382921 | 8.35 | 20.4 | 1677213(35.4%) |
| *S*. *castanea* | MYC1 | 6242996 | 101166 | 188 | 6141642 | 63.54 | 54.15 | 2985391(49.4%) |
|  | MYC2 | 2051644 | 14092 | 57 | 2037495 | 56.66 | 47.78 | 1119229(55.3%) |
|  | MYC3 | 10570974 | 42281 | 330 | 10528363 | 62.54 | 60.8 | 4739215(45.2%) |
|  | MYC4 | 1423842 | 13115 | 39 | 1410688 | 10.26 | 19.64 | 2277058(44.2%) |
|  | MYC5 | 3302668 | 17502 | 105 | 3285061 | 57.97 | 50.62 | 1651796(50.5%) |
|  | MYC6 | 4817272 | 39674 | 146 | 4777452 | 56.71 | 49.07 | 2518791(53.2%) |
|  | MYC7 | 2166124 | 12109 | 59 | 2153956 | 54.12 | 46.53 | 1138632(53.1%) |
|  | MYC8 | 1634632 | 11025 | 40 | 1623567 | 48.92 | 48.94 | 887131(54.2%) |
|  | MYC9 | 9119044 | 50056 | 297 | 9068691 | 64.27 | 62.47 | 4262219(47.2%) |
|  | MYC10 | 2414132 | 15226 | 73 | 2398833 | 26.14 | 42.15 | 1502211(47.6%) |
|  | MYC11 | 835104 | 40776 | 46 | 794282 | 8.88 | 17.69 | 2687618(43.2%) |
|  | MYC12 | 37683872 | 353634 | 1189 | 37329049 | 78.31 | 89.14 | 13701560(37.0%) |
|  | MYC13 | 1327770 | 13543 | 35 | 1314192 | 44.61 | 45.41 | 721186(54.2%) |
|  | MYC15 | 958316 | 12925 | 34 | 945357 | 16.26 | 29.98 | 637658(47.9%) |
|  | MYC16 | 16614228 | 142551 | 1958 | 16469719 | 90.97 | 85.42 | 6779538(41.5%) |
|  | MYC17 | 1288358 | 13210 | 172 | 1274976 | 9.71 | 22.27 | 1656521(44.8%) |
|  | MYC18 | 2130330 | 44597 | 104 | 2085629 | 19.35 | 52.37 | 1033272(40.4%) |
|  | MYC19 | 11775500 | 83515 | 1400 | 11690585 | 79.25 | 76.69 | 4895791(42.2%) |
|  | MYC20 | 10187916 | 79978 | 1179 | 10106759 | 70.42 | 72.63 | 4253772(42.4%) |
|  | MYC21 | 18994640 | 116029 | 2240 | 18876371 | 77.35 | 82.23 | 7090716(37.8%) |
|  | MYC22 | 7559740 | 102336 | 900 | 7456504 | 70.06 | 67.29 | 3265416(44.4%) |
|  | MYC23 | 18121216 | 156130 | 2152 | 17962934 | 74.18 | 73.24 | 7935957(44.6%) |
|  | MYC24 | 20173332 | 341358 | 688 | 19831286 | 118.96 | 114.26 | 7296517(37.2%) |
|  | MYC25 | 11494274 | 70494 | 1356 | 11422424 | 89.76 | 81.47 | 4947073(43.6%) |
|  | MYC26 | 7898522 | 89488 | 276 | 7808758 | 113.39 | 101.67 | 3271510(42.4%) |
|  | MYC27 | 4966356 | 52178 | 155 | 4914023 | 97.33 | 90.55 | 2146430(44.1%) |
|  | MYC28 | 13142606 | 88101 | 1522 | 13052983 | 89.31 | 86.04 | 5388254(41.6%) |
|  | MYC29 | 18963796 | 180585 | 593 | 18782618 | 110.6 | 113.41 | 6409070(34.4%) |
|  | MYC30 | 13987478 | 130368 | 1740 | 13855370 | 90.63 | 87.25 | 6006421(43.8%) |
| putative hybrids | MYH1 | 5252926 | 27333 | 3215 | 5222378 | 63.07 | 58.97 | 2825644(54.4%) |
|  | MYH2 | 9689930 | 84835 | 672 | 9604423 | 97.41 | 92.4 | 3982308(41.8%) |
|  | MYH5 | 6157312 | 43221 | 3720 | 6110371 | 63.42 | 59.3 | 2992526(49.3%) |
|  | MYH6 | 11723286 | 77905 | 7219 | 11638162 | 66.21 | 64.11 | 5307924(45.9%) |
|  | MYH7 | 10021866 | 50727 | 6302 | 9964837 | 65.25 | 63.68 | 4621255(46.6%) |
|  | MYH8 | 1800446 | 15348 | 1126 | 1783972 | 61.74 | 54.34 | 995035(56.3%) |
|  | MYH10 | 4005862 | 19365 | 2544 | 3983953 | 64.54 | 59.4 | 2014087(50.8%) |
|  | MYH12 | 1273862 | 10406 | 818 | 1262638 | 60.64 | 52.64 | 764600(61.0%) |
|  | MYH13 | 5150128 | 29668 | 3045 | 5117415 | 64.32 | 58.93 | 2761060(54.3%) |
|  | MYH14 | 9298372 | 110273 | 628 | 9187471 | 100.43 | 116.05 | 3410666(37.6%) |
|  | MYH15 | 5511006 | 33582 | 3410 | 5474014 | 65.02 | 59.69 | 2923573(53.7%) |
|  | MYH18 | 4029340 | 52348 | 125 | 3976867 | 67.49 | 67.08 | 1815335(46.1%) |
|  | MYH19 | 2593960 | 17137 | 86 | 2576737 | 66.34 | 64.57 | 1267214(49.5%) |
|  | MYH20 | 13052452 | 88967 | 396 | 12963089 | 59.22 | 67.79 | 5154159(40.0%) |
|  | MYH21 | 23073996 | 130701 | 637 | 22942658 | 61.31 | 69.6 | 9555228(41.9%) |
|  | MYH22 | 1490338 | 27606 | 49 | 1462683 | 57.74 | 55.57 | 722495(50.3%) |
|  | MYH23 | 6624138 | 45776 | 212 | 6578150 | 67.01 | 69.41 | 2895891(44.3%) |
|  | MYH24 | 4826616 | 23948 | 155 | 4802513 | 63.86 | 64.17 | 2187949(45.8%) |
|  | MYH26 | 1785918 | 9901 | 51 | 1775966 | 76.04 | 70.55 | 909227(51.5%) |
|  | MYH27 | 2835242 | 34477 | 86 | 2800679 | 122.22 | 98.16 | 1402514(50.7%) |
|  | MYH54 | 4428168 | 35707 | 28 | 4392433 | 44.23 | 44.95 | 2250851(51.7%) |
|  | MYH56 | 2385546 | 151367 | 152 | 2234027 | 60.56 | 62.12 | 1023541(48.7%) |
|  | MYH59 | 3736126 | 290612 | 206 | 3445308 | 68.53 | 62.64 | 1843658(56.6%) |
|  | MYH61 | 8094824 | 44868 | 61 | 8049895 | 46.5 | 49.92 | 3998852(49.9%) |
|  | MYH66 | 17098828 | 89418 | 116 | 17009294 | 53.03 | 61.55 | 7716246(45.6%) |
|  | MYH68 | 1193284 | 9225 | 7 | 1184052 | 36.21 | 35.96 | 661431(55.6%) |
|  | MYH77 | 998008 | 59817 | 17 | 938174 | 10.65 | 21.8 | 662889(42.2%) |
|  | MYH87 | 4009134 | 24399 | 27 | 3984708 | 44.31 | 43.86 | 2015377(50.9%) |
|  | MYH90 | 850826 | 12787 | 3 | 838036 | 8.96 | 18.44 | 1028071(45.3%) |
|  | MYH118 | 11355074 | 178316 | 226 | 11176532 | 14.62 | 52.71 | 1852560(14.6%) |
|  | MYH120 | 12106078 | 85250 | 402 | 12020426 | 95.72 | 131.44 | 2426298(20.3%) |
|  | MYH135 | 17185532 | 102534 | 527 | 17082471 | 100.25 | 143.18 | 3254315(19.2%) |
|  | MYH148 | 1180180 | 106798 | 29 | 1073353 | 7.89 | 17.59 | 4333381(40.1%) |
|  | MYH174 | 48045092 | 807398 | 3042 | 47234652 | 78.83 | 83.37 | 20379060(43.8%) |
|  | MYH186 | 2476636 | 817452 | 83 | 1659101 | 11.24 | 25.31 | 1448922(44.5%) |
|  | MYH255 | 11757798 | 177511 | 761 | 11579526 | 67.94 | 70.44 | 5370324(47.1%) |
|  | MYH347 | 22876948 | 150045 | 705 | 22726198 | 141.33 | 184.5 | 5070605(22.5%) |

**Supplementary Table 5.** Pairwise *F*_ST_ values for *S*. *flava*, *S*. *castane* and the putative hybrids at the MY location based on 2,216 SNPs

|  | *S. flava* | Putative hybrids | *S. castanea* |
| --- | --- | --- | --- |
| *S. flava* | * | 0.0520528 | 0.138486 |
| Putative hybrids | 0.0520528 | * | 0.0465692 |
| *S. castanea* | 0.138486 | 0.0465692 | * |

**Supplementary Table 6.** Pairwise *F*_ST_ values for *S*. *flava*, *S*. *castane* and the putative hybrids at the HSH location based on 3,895 SNPs

|  | *S. flava* | Putative hybrids | *S. castanea* |
| --- | --- | --- | --- |
| *S. flava* | * | 0.187327 | 0.257635 |
| Putative hybrids | 0.187327 | * | 0.152179 |
| *S. castanea* | 0.257635 | 0.152179 | * |

**Supplementary Table 7.** Volatile compounds extracted from flowers of *S*. *flava*, *S*. *castanea* and hybrids (n = 3) at the MY location

| Compound | CAS | Mean ± S.D. (%) | | |
| --- | --- | --- | --- | --- |
|  |  | *S*. *flava* | *S*. *castanea* | Putative hybrids |
| **Monoterpenoids** |  |  |  |  |
| 3-thujene | 002867-05-2 | 2.42 ± 0.2696 | 2.81 ± 0.0577 | 1.76 ± 0.0586 |
| α-pinene | 000080-56-8 | 7.27 ± 1.1442 | 12.89 ± 0.4850 | 9.70 ± 0.2554 |
| β-pinene | 000127-91-3 | 22.37 ± 2.9187 | 8.53 ± 0.4215 | 19.64 ± 0.6384 |
| sabenene | 003387-41-5 | 4.32 ± 0.1587 | 5.35 ± 0.3406 | 7.28 ± 0.3350 |
| β-phellandrene | 000555-10-2 | 14.03 ± 0.2862 | 6.83 ± 0.5369 | 23.57 ± 1.1558 |
| 1,3-cyclohexadiene | 000099-86-5 | 1.11 ± 0.0854 | — | 0.77 ± 0.0577 |
| γ-terpinene | 000099-85-4 | 6.08± 0.4193 | 1.86 ± 0.0058 | 2.38 ± 0.1212 |
| camphene | 000079-92-5 | — | 16.60 ± 0.4619 | 5.34 ± 0.1882 |
| myrcene | 000123-35-3 | — | 2.24 ± 0.0924 | 1.24 ± 0.1931 |
| 3-carene | 013466-78-9 | — | 1.07 ± 0.0693 | 3.01 ± 0.5116 |
| ocimene | 013877-91-3 | — | — | 0.56 ± 0.1210 |
| trans-β-ocimene | 003779-61-1 | 3.68 ± 0.0513 | — | 3.23 ± 0.3408 |
| camphor | 000076-22-2 | — | 4.90 ± 0.0115 | 0.79 ± 0.0404 |
| borneol | 000507-70-0 | — | 3.81 ± 0.1155 | — |
| **Sesquiterpenes** |  |  |  |  |
| β-bourbonene | 005208-59-3 | 0.98 ± 0.1582 | — | 0.82 ± 0.1401 |
| α- caryophyllene | 006753-98-6 | 1.49 ± 0.1966 | — | 0.90 ± 0.0416 |
| β- caryophyllene | 000087-44-5 | 2.22 ± 0.2309 | 0.65 ± 0.0924 | 1.47 ± 0.0458 |
| germacrene D | 023986-74-5 | 1.52 ± 0.1815 | 0.54 ± 0.0000 | 0.79 ± 0.0473 |
| δ-cadinene | 000483-76-1 | 0.67 ± 0.0985 | 0.56 ± 0.0000 | 0.47 ± 0.0520 |
| **Aromatics** |  |  |  |  |
| m-cymene | 000535-77-3 | 2.13 ± 0.1595 | — | — |
| p-cymene | 000099-87-6 | — | 1.40 ± 0.2194 | 1.86 ± 0.0961 |
| **Aliphatics** |  |  |  |  |
| 2,2,4,6,6-pentamethyl-heptane | 013475-82-6 | 10.05 ± 0.9318 | 10.49 ± 1.4318 | 3.64 ± 0.0874 |

**Supplementary Table 8.** Fruit set and seed numbers per fruit from inter- and intra-specific pollinations of *Salvia* species in this study at MY location

| Treatments  (n = 35) | Fruit set  (Mean ± S.E.%) | P value | Z | Seeds per fruit  (Mean ± S.E.) | P value | Z |
| --- | --- | --- | --- | --- | --- | --- |
| *S*. *flava* × *S*. *flava* | 80.00 ± 6.86 |  |  | 2.3429 ± 0.2623 |  |  |
| *S*. *flava* × *S*. *castanea* | 80.00 ± 6.86 |  |  | 2.0571 ± 0.2525 |  |  |
| *S*. *castanea* × *S*. *flava* | 57.14 ± 8.49 |  |  | 1.9714 ± 0.3188 |  |  |
| *S*. *castanea* × *S*. *castanea* | 60.00 ± 8.40 |  |  | 2.1143 ± 0.3169 |  |  |
| hybrids × hybrids (geitonogamy) | 48.57 ± 8.57 | 0.634 | –0.476 | 0.9429 ± 0.2167 | 0.851 | *–*0.187 |
| hybrids × hybrids (xenogamy) | 42.86 ± 8.49 |  |  | 1.0286 ± 0.2576 |  |  |

**Supplementary Table 9.** Multifactor Analysis of Variance for fruit set and seed numbers per fruit from pollination treatments between *S.* *flava* and *S*. *castanea* at MY location

| Source | Fruit set | | Seed number per fruit | |
| --- | --- | --- | --- | --- |
|  | F | P value | F | P value |
| Mother species | 7.759 | **0.006** | 0.295 | 0.588 |
| Cross type | 0.034 | 0.853 | 0.549 | 0.460 |
| Mother species × Cross type | 0.034 | 0.853 | 0.061 | 0.85 |
